# 2-Chloro-3-[(*E*)-(hydrazin-1-yl­idene)meth­yl]-6-meth­oxy­quinoline

**DOI:** 10.1107/S1600536812017977

**Published:** 2012-04-28

**Authors:** Sofiane Bouacida, Abdelmalek Bouraiou, Nassima Benhamoud, Thierry Roisnel, Ali Belfaitah

**Affiliations:** a Unité de Recherche de Chimie de l’Environnement et Moléculaire Structurale (CHEMS), Université Mentouri-Constantine, 25000 Algeria; b Laboratoire des Produits Naturels d’Origine Végétale et de Synthèse Organique, PHYSYNOR, Université Mentouri-Constantine, 25000 Constantine, Algeria; c Centre de Difractométrie X, UMR 6226 CNRS Unité Sciences Chimiques de Rennes, Université de Rennes I, 263 Avenue du Général Leclerc, 35042 Rennes, France

## Abstract

In the title compound, C_11_H_10_ClN_3_O, the quinoline ring system is essentially planar, the r.m.s. deviation for the non-H atoms being 0.014 (2) Å with a maximum deviation from the mean plane of 0.0206 (14) Å for the C atom bonded to the –CH—N=NH_2_ group. In the crystal, molecules are linked *via* N—H⋯O and N—H⋯N hydrogen bonds, forming zigzag layers parallel to (010).

## Related literature

For previous work on mol­ecules with a quinolyl moiety, see: Benzerka *et al.* (2011 ▶); Belfaitah *et al.* (2006 ▶) Bouraiou *et al.* (2008 ▶, 2011 ▶); Ladraa *et al.* (2009 ▶). For applications of pyrazole and its derivatives, see: Mali *et al.* (2010 ▶); Paul *et al.* (2001 ▶).
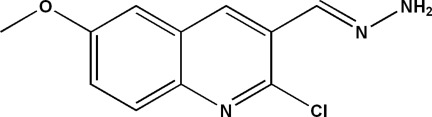


## Experimental

### 

#### Crystal data

C_11_H_10_ClN_3_O
*M* *_r_* = 235.67
Orthorhombic, 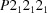

*a* = 3.8949 (2) Å
*b* = 12.0510 (5) Å
*c* = 21.9910 (9) Å
*V* = 1032.20 (8) Å^3^
*Z* = 4
Mo *K*α radiation
μ = 0.35 mm^−1^
*T* = 150 K
0.28 × 0.15 × 0.14 mm

#### Data collection

Bruker APEXII diffractometer
Absorption correction: multi-scan (*SADABS*; Sheldrick, 2002 ▶) *T*_min_ = 0.898, *T*_max_ = 0.952
15777 measured reflections
2352 independent reflections
2044 reflections with *I* > 2σ(*I*)
*R*_int_ = 0.044

#### Refinement

*R*[*F* ^2^ > 2σ(*F* ^2^)] = 0.032
*wR*(*F* ^2^) = 0.073
*S* = 1.06
2352 reflections
147 parameters
H-atom parameters constrained
Δρ_max_ = 0.31 e Å^−3^
Δρ_min_ = −0.26 e Å^−3^
Absolute structure: Flack (1983 ▶), 922 Friedel pairs
Flack parameter: 0.00 (6)

### 

Data collection: *APEX2* (Bruker, 2004 ▶); cell refinement: *SAINT* (Bruker, 2004 ▶); data reduction: *SAINT* (Bruker, 2004 ▶); program(s) used to solve structure: *SIR2002* (Burla *et al.*, 2003 ▶); program(s) used to refine structure: *SHELXL97* (Sheldrick, 2008 ▶); molecular graphics: *ORTEP-3 for Windows* (Farrugia, 1997 ▶) and *DIAMOND* (Brandenburg & Berndt, 2001 ▶); software used to prepare material for publication: *WinGX* (Farrugia, 1999 ▶).

## Supplementary Material

Crystal structure: contains datablock(s) global, I. DOI: 10.1107/S1600536812017977/fj2545sup1.cif

Structure factors: contains datablock(s) I. DOI: 10.1107/S1600536812017977/fj2545Isup2.hkl

Supplementary material file. DOI: 10.1107/S1600536812017977/fj2545Isup3.cml

Additional supplementary materials:  crystallographic information; 3D view; checkCIF report

## Figures and Tables

**Table 1:** Hydrogen-bond geometry (Å, °)

*D*—H⋯*A*	*D*—H	H⋯*A*	*D*⋯*A*	*D*—H⋯*A*
N13—H13*A*⋯O14^i^	0.88	2.34	3.219 (2)	178
N13—H13*B*⋯N13^ii^	0.88	2.19	3.058 (2)	169

Symmetry codes: (i) 
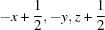
; (ii) 
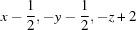
.

**Fig. 1.**
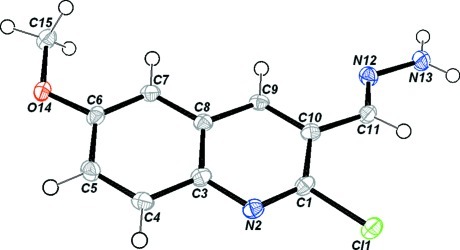
(Farrugia, 1997) the structure of the title compound with the atomic labelling scheme. Displacement are drawn at the 50% probability level.

**Fig. 2.**
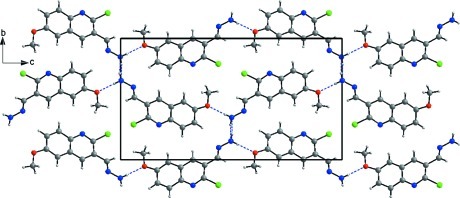
(Brandenburg & Berndt, 2001) A diagram of the layered crystal packing of (I) viewed down the a axis and showing hydrogen bond [N—H···O and N—H···N] as dashed line.

## supplementary crystallographic information

### Comment

Pyrazole and its derivatives are gaining importance in medicinal and organic chemistry. They have displayed broad spectrum of pharmacological and biological activities such as anti-bacterial, anti-depressant, and anti-hyperglycemic (Mali *et al.*, 2010). Pyrazolo[3,4-*b*]quinolines have displayed bioactivities such as antiviral, antimalarial, lowering of serum cholesterol (Paul *et al.*, 2001), but no metal complexes of such drugs have been reported in the past which might possibly have better pharmaceutical effect. Therefore, studies of the metal complexes are important in the search for new drugs. In previous works, we were interested in the design and synthesis of new molecules that contain a quinolyl moiety (Belfaitah *et al.*, 2006; Bouraiou *et al.*, 2008, 2011; Ladraa *et al.*, 2009 and Benzerka *et al.*, 2011). In this paper, we report the structure determination of compound resulting from an unwanted reaction of the 6-methoxy-1*H*-pyrazolo[3,4-*b*]quinoline with RuCl_3_ in acidic conditions. Our attempt to synthesis the pyrazolo[3,4-*b*]quinoline/Ruthenium complex was failed and led to (*E*)-1-((2-chloro-6-methylquinolin-3-yl)methylene)hydrazine (I).

The molecular geometry and the atom-numbering scheme of (I) are shown in Fig. 1. In the asymetric unit of title molecule, (C_11_ H_10_ Cl N_3_ O), the chloro-quinolyl unit is linked to methoxy and methylenehydrazine group. The quinoline ring system is essentially planar; the r.m.s. deviation for the non-H atoms is 0.014 (2) Å with a maximum deviation from the mean plane of 0.0206 (14) Å for the C atom bonded to the –CH—N═NH_2_ group. The crystal packing can be described as layers in zigzag parallel to the (010) plane (Fig. 2). It is stabilized by N—H···O and N—H···N intermolecular hydrogen bonds (Fig. 2). These interaction bonds link the molecules within the layers and also link the layers together, reinforcing the cohesion of the structure. Hydrogen-bonding parameters are listed in table 1.

### Experimental

First, 6-methoxy-1*H*-pyrazolo[3,4-*b*]quinoline was prepared from 2-chloro-6-methoxyquinoline-3-carbaldehyde and hydrazine hydrate in refluxing ethanol in a one-pot synthesis. Next, a mixture of 6-methoxy-1*H*-pyrazolo[3,4-*b*]quinoline(5 mmol)and RuCl_3_(5 mmol) in aqueous HCl(10 ml) was stirred at 50°C for 1 h. Under these conditions, compound I was successfully obtained. Single crystals suitable for X-ray diffraction analysis were obtained by dissolving the corresponding compound in methanol solution and letting it for slow evaporation at room temperature.

### Refinement

All non-H atoms were refined with anisotropic atomic displacement parameters. All H atoms were localized on Fourier maps but introduced in calculated positions and treated as riding on their parent C or N atom. (with C—H = 0.95 and 0.98 Å, N—H = 0.88 Å and *U*_iso_(H) =1.5 or 1.2(carrier atom)).

### Crystal data

**Table:**

C_11_H_10_ClN_3_O	*D*_x_ = 1.517 Mg m^−^^3^
*M**_r_* = 235.67	Mo *K*α radiation, λ = 0.71073 Å
Orthorhombic, *P*2_1_2_1_2_1_	Cell parameters from 26476 reflections
*a* = 3.8949 (2) Å	θ = 2.9–27.5°
*b* = 12.0510 (5) Å	µ = 0.35 mm^−^^1^
*c* = 21.9910 (9) Å	*T* = 150 K
*V* = 1032.20 (8) Å^3^	Prism, colourless
*Z* = 4	0.28 × 0.15 × 0.14 mm
*F*(000) = 488	

### Data collection

**Table:**

Bruker APEXII diffractometer	2352 independent reflections
Radiation source: Enraf–Nonius FR590	2044 reflections with *I* > 2σ(*I*)
Graphite monochromator	*R*_int_ = 0.044
Detector resolution: 9 pixels mm^-1^	θ_max_ = 27.5°, θ_min_ = 3.3°
CCD rotation images, thin slices scans	*h* = −5→5
Absorption correction: multi-scan (*SADABS*; Sheldrick, 2002)	*k* = −15→15
*T*_min_ = 0.898, *T*_max_ = 0.952	*l* = −28→28
15777 measured reflections	

### Refinement

**Table:**

Refinement on *F*^2^	Secondary atom site location: difference Fourier map
Least-squares matrix: full	Hydrogen site location: inferred from neighbouring sites
*R*[*F*^2^ > 2σ(*F*^2^)] = 0.032	H-atom parameters constrained
*wR*(*F*^2^) = 0.073	*w* = 1/[σ^2^(*F*_o_^2^) + (0.0395*P*)^2^ + 0.2196*P*] where *P* = (*F*_o_^2^ + 2*F*_c_^2^)/3
*S* = 1.06	(Δ/σ)_max_ = 0.001
2352 reflections	Δρ_max_ = 0.31 e Å^−^^3^
147 parameters	Δρ_min_ = −0.26 e Å^−^^3^
0 restraints	Absolute structure: Flack (1983), 922 Friedel pairs
Primary atom site location: structure-invariant direct methods	Flack parameter: 0.00 (6)

### Special details

**Table:**

Geometry. All e.s.d.'s (except the e.s.d. in the dihedral angle between two l.s. planes) are estimated using the full covariance matrix. The cell e.s.d.'s are taken into account individually in the estimation of e.s.d.'s in distances, angles and torsion angles; correlations between e.s.d.'s in cell parameters are only used when they are defined by crystal symmetry. An approximate (isotropic) treatment of cell e.s.d.'s is used for estimating e.s.d.'s involving l.s. planes.
Refinement. Refinement of *F*^2^ against ALL reflections. The weighted *R*-factor *wR* and goodness of fit *S* are based on *F*^2^, conventional *R*-factors *R* are based on *F*, with *F* set to zero for negative *F*^2^. The threshold expression of *F*^2^ > σ(*F*^2^) is used only for calculating *R*-factors(gt) *etc*. and is not relevant to the choice of reflections for refinement. *R*-factors based on *F*^2^ are statistically about twice as large as those based on *F*, and *R*- factors based on ALL data will be even larger.

### Fractional atomic coordinates and isotropic or equivalent isotropic displacement parameters (Å^2^)

**Table:**

	*x*	*y*	*z*	*U*_iso_*/*U*_eq_	
C1	0.4493 (5)	0.15128 (15)	0.87535 (8)	0.0170 (4)	
C3	0.3425 (5)	0.16948 (14)	0.77399 (8)	0.0168 (4)	
C4	0.3615 (5)	0.23375 (14)	0.72037 (8)	0.0187 (4)	
H4	0.4634	0.3053	0.7216	0.022*	
C5	0.2342 (5)	0.19378 (14)	0.66667 (8)	0.0201 (4)	
H5	0.2468	0.2378	0.6309	0.024*	
C6	0.0840 (5)	0.08694 (15)	0.66430 (8)	0.0179 (4)	
C7	0.0598 (5)	0.02243 (15)	0.71528 (8)	0.0173 (4)	
H7	−0.0414	−0.0492	0.7131	0.021*	
C8	0.1864 (5)	0.06299 (13)	0.77136 (8)	0.0160 (4)	
C9	0.1641 (5)	0.00226 (13)	0.82624 (8)	0.0156 (4)	
H9	0.0595	−0.069	0.8261	0.019*	
C10	0.2916 (5)	0.04468 (14)	0.87986 (8)	0.0165 (4)	
C11	0.2622 (5)	−0.01392 (14)	0.93795 (8)	0.0179 (4)	
H11	0.3884	0.0114	0.9722	0.021*	
C15	−0.1868 (5)	−0.05136 (14)	0.60289 (8)	0.0219 (4)	
H15A	−0.0198	−0.1085	0.6143	0.033*	
H15B	−0.2623	−0.0637	0.5609	0.033*	
H15C	−0.3852	−0.0552	0.6302	0.033*	
N2	0.4745 (4)	0.21203 (13)	0.82682 (6)	0.0176 (3)	
N12	0.0681 (4)	−0.09888 (12)	0.94285 (7)	0.0202 (3)	
N13	0.0692 (5)	−0.15224 (13)	0.99808 (7)	0.0240 (4)	
H13A	0.1995	−0.1275	1.0278	0.029*	
H13B	−0.0603	−0.2112	1.0037	0.029*	
Cl1	0.62727 (12)	0.20978 (3)	0.941458 (19)	0.02055 (12)	
O14	−0.0299 (4)	0.05616 (10)	0.60762 (5)	0.0210 (3)	

### Atomic displacement parameters (Å^2^)

**Table:**

	*U*^11^	*U*^22^	*U*^33^	*U*^12^	*U*^13^	*U*^23^
C1	0.0146 (10)	0.0199 (9)	0.0167 (8)	0.0006 (7)	0.0013 (8)	−0.0050 (7)
C3	0.0146 (9)	0.0175 (8)	0.0183 (8)	0.0019 (8)	0.0024 (8)	−0.0024 (7)
C4	0.0198 (9)	0.0143 (8)	0.0219 (8)	0.0004 (8)	0.0038 (8)	0.0006 (7)
C5	0.0244 (10)	0.0191 (9)	0.0170 (9)	0.0024 (8)	0.0041 (8)	0.0025 (7)
C6	0.0179 (10)	0.0207 (9)	0.0151 (8)	0.0033 (8)	0.0011 (8)	−0.0031 (7)
C7	0.0193 (10)	0.0138 (8)	0.0189 (9)	−0.0001 (7)	0.0025 (8)	−0.0011 (7)
C8	0.0149 (10)	0.0155 (8)	0.0176 (8)	0.0032 (7)	0.0025 (8)	−0.0012 (7)
C9	0.0158 (10)	0.0121 (8)	0.0188 (8)	−0.0007 (8)	0.0031 (8)	0.0000 (7)
C10	0.0149 (10)	0.0163 (9)	0.0184 (9)	0.0027 (7)	0.0024 (7)	−0.0020 (7)
C11	0.0190 (9)	0.0191 (9)	0.0156 (8)	0.0017 (7)	−0.0014 (8)	−0.0031 (8)
C15	0.0242 (11)	0.0227 (9)	0.0187 (9)	−0.0015 (8)	−0.0010 (9)	−0.0039 (7)
N2	0.0173 (7)	0.0173 (7)	0.0181 (7)	−0.0005 (7)	0.0025 (6)	−0.0021 (7)
N12	0.0224 (8)	0.0201 (7)	0.0181 (7)	0.0014 (6)	0.0024 (8)	0.0018 (7)
N13	0.0339 (10)	0.0203 (8)	0.0177 (7)	−0.0039 (8)	−0.0013 (8)	0.0034 (6)
Cl1	0.0223 (2)	0.0211 (2)	0.01815 (19)	−0.00307 (19)	−0.0007 (2)	−0.00380 (18)
O14	0.0302 (8)	0.0194 (7)	0.0135 (6)	−0.0021 (5)	−0.0012 (6)	−0.0008 (5)

### Geometric parameters (Å, º)

**Table:**

C1—N2	1.298 (2)	C8—C9	1.414 (2)
C1—C10	1.428 (2)	C9—C10	1.378 (2)
C1—Cl1	1.7581 (17)	C9—H9	0.95
C3—N2	1.370 (2)	C10—C11	1.464 (2)
C3—C4	1.413 (2)	C11—N12	1.277 (2)
C3—C8	1.421 (2)	C11—H11	0.95
C4—C5	1.368 (3)	C15—O14	1.436 (2)
C4—H4	0.95	C15—H15A	0.98
C5—C6	1.415 (3)	C15—H15B	0.98
C5—H5	0.95	C15—H15C	0.98
C6—C7	1.368 (2)	N12—N13	1.374 (2)
C6—O14	1.374 (2)	N13—H13A	0.88
C7—C8	1.415 (2)	N13—H13B	0.88
C7—H7	0.95		
			
N2—C1—C10	126.68 (16)	C10—C9—C8	121.08 (15)
N2—C1—Cl1	115.08 (13)	C10—C9—H9	119.5
C10—C1—Cl1	118.23 (13)	C8—C9—H9	119.5
N2—C3—C4	118.87 (16)	C9—C10—C1	115.43 (15)
N2—C3—C8	122.21 (15)	C9—C10—C11	122.66 (15)
C4—C3—C8	118.92 (16)	C1—C10—C11	121.89 (15)
C5—C4—C3	120.56 (16)	N12—C11—C10	120.43 (17)
C5—C4—H4	119.7	N12—C11—H11	119.8
C3—C4—H4	119.7	C10—C11—H11	119.8
C4—C5—C6	120.12 (16)	O14—C15—H15A	109.5
C4—C5—H5	119.9	O14—C15—H15B	109.5
C6—C5—H5	119.9	H15A—C15—H15B	109.5
C7—C6—O14	124.60 (16)	O14—C15—H15C	109.5
C7—C6—C5	121.03 (16)	H15A—C15—H15C	109.5
O14—C6—C5	114.37 (15)	H15B—C15—H15C	109.5
C6—C7—C8	119.60 (16)	C1—N2—C3	117.24 (15)
C6—C7—H7	120.2	C11—N12—N13	116.60 (16)
C8—C7—H7	120.2	N12—N13—H13A	120
C9—C8—C7	122.92 (16)	N12—N13—H13B	120
C9—C8—C3	117.32 (16)	H13A—N13—H13B	120
C7—C8—C3	119.76 (16)	C6—O14—C15	116.53 (14)
			
N2—C3—C4—C5	179.61 (17)	C8—C9—C10—C1	−1.1 (3)
C8—C3—C4—C5	−0.5 (3)	C8—C9—C10—C11	177.74 (17)
C3—C4—C5—C6	−0.4 (3)	N2—C1—C10—C9	2.1 (3)
C4—C5—C6—C7	0.7 (3)	Cl1—C1—C10—C9	−178.57 (14)
C4—C5—C6—O14	−179.26 (17)	N2—C1—C10—C11	−176.71 (17)
O14—C6—C7—C8	179.99 (18)	Cl1—C1—C10—C11	2.6 (2)
C5—C6—C7—C8	0.1 (3)	C9—C10—C11—N12	−11.3 (3)
C6—C7—C8—C9	178.61 (17)	C1—C10—C11—N12	167.39 (17)
C6—C7—C8—C3	−1.0 (3)	C10—C1—N2—C3	−1.3 (3)
N2—C3—C8—C9	1.4 (3)	Cl1—C1—N2—C3	179.38 (13)
C4—C3—C8—C9	−178.41 (18)	C4—C3—N2—C1	179.25 (17)
N2—C3—C8—C7	−178.88 (17)	C8—C3—N2—C1	−0.6 (3)
C4—C3—C8—C7	1.3 (3)	C10—C11—N12—N13	176.74 (15)
C7—C8—C9—C10	179.82 (18)	C7—C6—O14—C15	0.5 (3)
C3—C8—C9—C10	−0.5 (3)	C5—C6—O14—C15	−179.58 (15)

### Hydrogen-bond geometry (Å, º)

**Table:**

*D*—H···*A*	*D*—H	H···*A*	*D*···*A*	*D*—H···*A*
N13—H13*A*···O14^i^	0.88	2.34	3.219 (2)	178
N13—H13*B*···N13^ii^	0.88	2.19	3.058 (2)	169
C11—H11···Cl1	0.95	2.65	3.0488 (18)	106

Symmetry codes: (i) −*x*+1/2, −*y*, *z*+1/2; (ii) *x*−1/2, −*y*−1/2, −*z*+2.
